# Utilizing collateral effects of antibiotic resistance to sustain antibiotic efficacy and addressing current barriers to translation

**DOI:** 10.1099/mic.0.001795

**Published:** 2026-11-10

**Authors:** Sidra Kashif, Beth A. James, Maria Rosa Domingo-Sananes, Alasdair T. M. Hubbard

**Affiliations:** 1 Department of Biosciences, Nottingham Trent University, Clifton Campus, College Drive, Clifton, Nottingham, NG11 8NS, UK; 2 Department of Clinical Sciences, Liverpool School of Tropical Medicine, Pembroke Place, Liverpool, L3 5QA, UK; 3 School of Veterinary Medicine and Sciences, University of Nottingham, Sutton Bonington Campus, Sutton Bonington, Leicestershire, LE12 5RD, UK

**Keywords:** antibiotic resistance, collateral effects, collateral susceptibility, epistasis, fitness, mutant selection windows

## Abstract

Antimicrobial resistance (AMR) results from acquiring mutations or genes that allow microbes to withstand antimicrobials. These genetic modifications can also cause non-selected changes in other cell phenotypes, such as fitness and susceptibility to antibiotic classes different from the one under selection. These collateral effects of AMR acquisition could be exploited to improve treatments. This is increasingly important in light of AMR as a major global health problem, associated with 4.71 million deaths worldwide in 2021, with 1.14 million deaths directly attributable to AMR. Additionally, the lack of development of novel antimicrobials active against resistant infections continues to compound the crisis. Even if new antimicrobials are developed, resistance will most likely emerge through adaptive evolution. Therefore, it is imperative to explore strategies to maintain the efficacy of new and existing antibiotics and limit the acquisition of AMR to continue to treat infections successfully. Understanding the impact of resistance acquisition on the bacterial phenotype, including collateral effects, has the potential to improve therapeutic outcomes. In this perspective, we will summarize the collateral effects of resistance acquisition, including fitness costs, collateral susceptibility networks, mutant selection windows and the probability of establishment of bacterial populations, and explore how these may be exploited to enhance the effectiveness of existing antibiotics. We will also discuss how challenges such as genetic background, polymicrobial infections and the environment are current barriers to the translation of these principles into the clinic.

## Introduction

The ability of micro-organisms to grow in the presence of an antimicrobial to which they were previously susceptible is termed *antimicrobial resistance* (AMR, Table 1) [1]. Antimicrobial-resistant infections caused by bacteria are a considerable threat to human health worldwide, as they lead to treatment failures that result in increased healthcare costs and mortality rates [2]. An estimated 4.71 million deaths were associated with antimicrobial-resistant bacterial infections globally in 2021, with 1.14 million directly attributed to AMR [3]. Deaths associated with AMR are predicted to rise to 8.22 million by 2050 [3]. AMR impacts not only healthcare but also the economy, with ~1% decrease in global gross domestic product predicted by 2050, which will be more acutely incurred in low- and middle-income countries with a predicted 5–7% loss [1]. Lower socioeconomic status and areas of deprivation [4] have been associated with a higher burden of AMR. A study in TX, USA, demonstrated that there was significant clustering of antimicrobial-resistant bacteria in areas associated with high levels of deprivation [4]. A systematic review identified that a higher risk of AMR and infectious disease in general was associated with several factors linked to socioeconomic status, including unemployment and poor living conditions [5]. The influence of socioeconomic status on AMR is of particular importance and concern in low- and middle-income countries, which are compounded by poor sanitation, education and access to healthcare [5–7].

**Table 1.** Glossary of key terms discussed in the review

Glossary	
AMR	Antimicrobial resistance
Antimicrobial stewardship	Systematic and coordinated approach to reduce AMR by promoting the responsible use of antimicrobials through education and support
Collateral effects	Phenotypic changes in bacteria, other than the canonical change in susceptibility, as a result of the acquisition of AMR
Collateral resistance	Resistance to one class of antibiotics confers resistance to other classes of antibiotics
Collateral susceptibility	Resistance to one class of antibiotics causes increased susceptibility to other classes of antibiotics
Compensatory mutation	A mutation that alleviates the deleterious effects of an AMR-conferring mutation
Epistasis	A genetic phenomenon where the phenotypic effect of a genetic variant depends on at least one other genetic variant in the genome; alternatively called genetic interactions or genetic background effects
Fitness	The ability of bacteria to survive, grow and reproduce in a given environment
Fitness costs	A reduction in the growth, reproduction and survival of a micro-organism (collectively termed fitness) due to a specific genetic change or adaptation
Genetic linkage	The propensity for genetic traits to be transferred together due to physical proximity in the DNA molecule
Minimum bactericidal concentration	The lowest concentration of an antimicrobial that kills 99.9% of a bacterial population
MIC	The lowest concentration of an antimicrobial that prevents visible growth of a specific micro-organism
Mutant prevention concentration	The minimum antimicrobial concentration that is required to prevent the selection of AMR mutants. Alternatively, it can be defined as the maximum concentration at which mutants can be selected for
Mutant selection window	The antimicrobial concentration range where single-step mutations increasing resistance can be selected for; it is defined as the concentration range between the MIC and the mutation prevention concentration (MPC)
Pleiotropic effects	A genetic phenomenon where variation at a single genetic locus affects multiple phenotypes
Population establishment probability	Probability of a single cell establishing a population

Bacteria acquire resistance to antimicrobials either through the acquisition of *de novo* mutations or AMR genes on mobile genetic elements (MGEs) via horizontal gene transfer (HGT). AMR mechanisms can result in reduced antimicrobial uptake, active antimicrobial efflux, modification of the antimicrobial target and modification/inactivation of the antimicrobial [8]. Although the acquisition of AMR in bacteria is a natural phenomenon, the overuse of antimicrobials in human, animal and agricultural settings accelerates selection for resistance and promotes the sharing of MGEs [9]. Several strategies [10–13] have been put forward to reduce AMR, such as *antimicrobial stewardship* (Table 1), rapid point-of-care diagnostics and development of novel antibiotics with varying success. Additionally, enhanced surveillance of diverse factors which are important in AMR, including antimicrobial use, antimicrobial consumption and incidence and spread of AMR pathogens, has been promoted. Multiple early-stage drug discovery projects are ongoing [14, 15], and several potential antimicrobials with novel targets that are unaffected by existing resistance mechanisms are entering the preclinical pipeline [16, 17]. Despite current drug development activity, the lack of economic incentives and a slow antimicrobial development pipeline has led to a discovery void, and pharmaceutical companies have deprioritized research and development of new antimicrobials. Therefore, non-antimicrobial treatment options have gained in popularity and are subject to intense research, including microbiome modulation [18], faecal microbiome transplant [19] and bacteriophage therapy [20].

Even with the potential development of new therapies, prolonging the useful life of antimicrobials currently in use is of high importance. This is the goal of antimicrobial stewardship, which aims to promote the judicious, evidence-based and optimized use of antimicrobials through support and education to limit the development of AMR. According to a recent meta-analysis, antimicrobial stewardship programmes have had considerable success, leading to a 10% overall reduction in prescriptions and 28% overall reduction in consumption [21]. The impact of antimicrobial stewardship is particularly evident in low- and middle-income countries and paediatric patients, but the degree of reduction is dependent on the antimicrobial, country, patient group and hospital setting [21]. Despite the success of antimicrobial stewardship, when assessing clinical audits, patient interviews and lack of indication of the need for antibiotics, inappropriate use of antibiotics is still high [22]. In high-, low- and middle-income countries, an average of ~30% antibiotics were inappropriately prescribed [22].

While the development of new point-of-care and rapid diagnostics has progressed over recent years, several limitations to widespread implementation remain, including the need for specialist expertise and knowledge to use and interpret the outcomes, mismatch between phenotype and genotype and cost [23]. Importantly, a meta-analysis found that rapid diagnostics alone did not reduce mortality; however, a combination of antimicrobial stewardship and rapid diagnostics did result in a significant decrease in mortality [24]. Although measures to counter AMR have been implemented at a global level, AMR is still rising. In recent years, an increased incidence of multidrug-resistant infections has been reported by the US Centers for Disease Control and Prevention, including a 50% increase in extended-spectrum *β*-lactamase-producing *Enterobacteriaceae* [25].

The World Health Organization (WHO) has reported that 170 countries have developed AMR national action plans (NAPs) [26, 27]. These span countries of all income levels and the reports consider diverse aspects to limit and reduce AMR, such as policy design, implementation tools and monitoring and evaluation [28]. Of the 166 countries that report progress to the WHO, 109 have already begun implementing their NAPs [27]. One area recommended for immediate research focus is the optimization of antimicrobial use and the selection of the most effective and appropriate antimicrobial to treat a specific infection. Keeping in view the ability of bacteria to evolve resistance, the necessity to use antimicrobials for treating infections and, with the scarcity of novel antibiotics, research on preserving the efficacy of existing antibiotics has rapidly increased in recent years [29].

One potential strategy to achieve this goal is to take advantage of the unintended impact of AMR acquisition on the bacterial phenotype. Acquisition of AMR by either mutation or HGT can affect phenotypic properties of a cell besides the acquired resistance, which are termed collateral effects. There are several excellent reviews on the underlying principles and mechanisms of collateral susceptibility and fitness costs of AMR [30–34]. In this perspective, we discuss several key collateral effects that have the potential to be exploited to limit the further acquisition of AMR and advocate for a holistic approach to their use and study. Additionally, we discuss how it may be possible to leverage these collateral effects to maintain the efficacy of current and novel antimicrobials and improve treatment outcomes, therefore enhancing antimicrobial stewardship. We focus on how the acquisition of resistance can result in changes in cell fitness, susceptibility to other antimicrobials, the capacity to evolve further resistance to alternative antimicrobials and the probability of establishing a population. We will also highlight barriers that can affect the translation of *in vitro* research into clinical strategies. We consider how the key properties identified above may vary in strains with different genetic backgrounds (due to epistatic interactions) and in different environments, including the contribution of other micro-organisms. While we focus on bacterial infections within this perspective, the principles can also be applied to some non-bacterial pathogens.

## Collateral effects

The acquisition of resistance to one antimicrobial can alter other cellular phenotypes, such as susceptibility to other antimicrobials and *fitness* (Table 1), which we termed *collateral effects* (Table 1), and constitute *pleiotropic effects* (Table 1) of AMR-conferring genes or mutations. Collateral effects may result from changes in cellular or metabolic processes and lead to trade-offs where the benefit of resistance to one antibiotic creates vulnerabilities that could be exploited to limit the emergence and spread of AMR [32, 35].

## Fitness

Fitness is defined as the reproductive success of an organism; in bacteria, it is based on the capacity to survive and reproduce [36]. It has often been proposed that acquisition of AMR, both through mutations and HGT, can lead to a significant decrease in fitness in the absence of the antimicrobial, termed *fitness costs* [37, 38] (Table 1). If this is the case, in the absence of the antimicrobial, susceptible populations should outcompete resistant bacteria [39] (Fig. 1). These fitness costs may be the result of mutations that impact important biological functions within the cell [40], such as in *Escherichia coli*, where mutations in the ribosomal protein S12 confer resistance to streptomycin but also have a negative effect on ribosomal regulation. Therefore, resistance mutations that impact the cellular function of the bacterial host can incur fitness costs when the environment is devoid of antibiotics. Alternatively, there may be a cost associated with maintaining and expressing horizontally acquired genes, such as the expression of the AMR gene, which may impose a fitness cost [41, 42]. For example, the plasmid pUHH239.2, which contains multiple AMR genes, including *bla*_CTX-M-15_, carried a 2.9% fitness cost in *E. coli* MG1655 [43]. The complete deletion of the entire resistance region on the plasmid abolished the fitness cost and the deletion of the region containing *bla*_CTX-M-15_ increased fitness by 1.5%. It was identified that *bla*_CTX-M-15_ was highly expressed compared to other genes, and when expression was increased further artificially, the fitness cost also increased [43]. Further analysis pinpointed that this fitness cost was due to the signal peptide of CTX-M-15 [43]. Fitness costs can also arise from interference of acquired genetic elements or gene variants with the bacteria’s cellular processes due to ‘genetic conflict’. For example, Hall *et al*. found that carriage of certain plasmids imposed a fitness cost resulting from plasmid-dependent changes in gene expression of host genes. However, fitness costs can be alleviated by *compensatory mutations* [44] (Table 1). The acquisition of compensatory mutations that ameliorate fitness costs complicates the use of fitness costs as a collateral effect. For example, mutations within *rpoA* and *rpoC* have been associated with compensation of the fitness cost of rifampicin-resistance in *Mycobacterium tuberculosis* [45]. These add another layer of complexity to harnessing fitness as a collateral effect to optimize antimicrobials. It is therefore important to assess the fitness costs of diverse mutations and AMR genes to a single antimicrobial and to test further evolution (in the presence and absence of the antimicrobial) to determine the impact of compensatory mutations [46, 47].

**Fig. 1.**
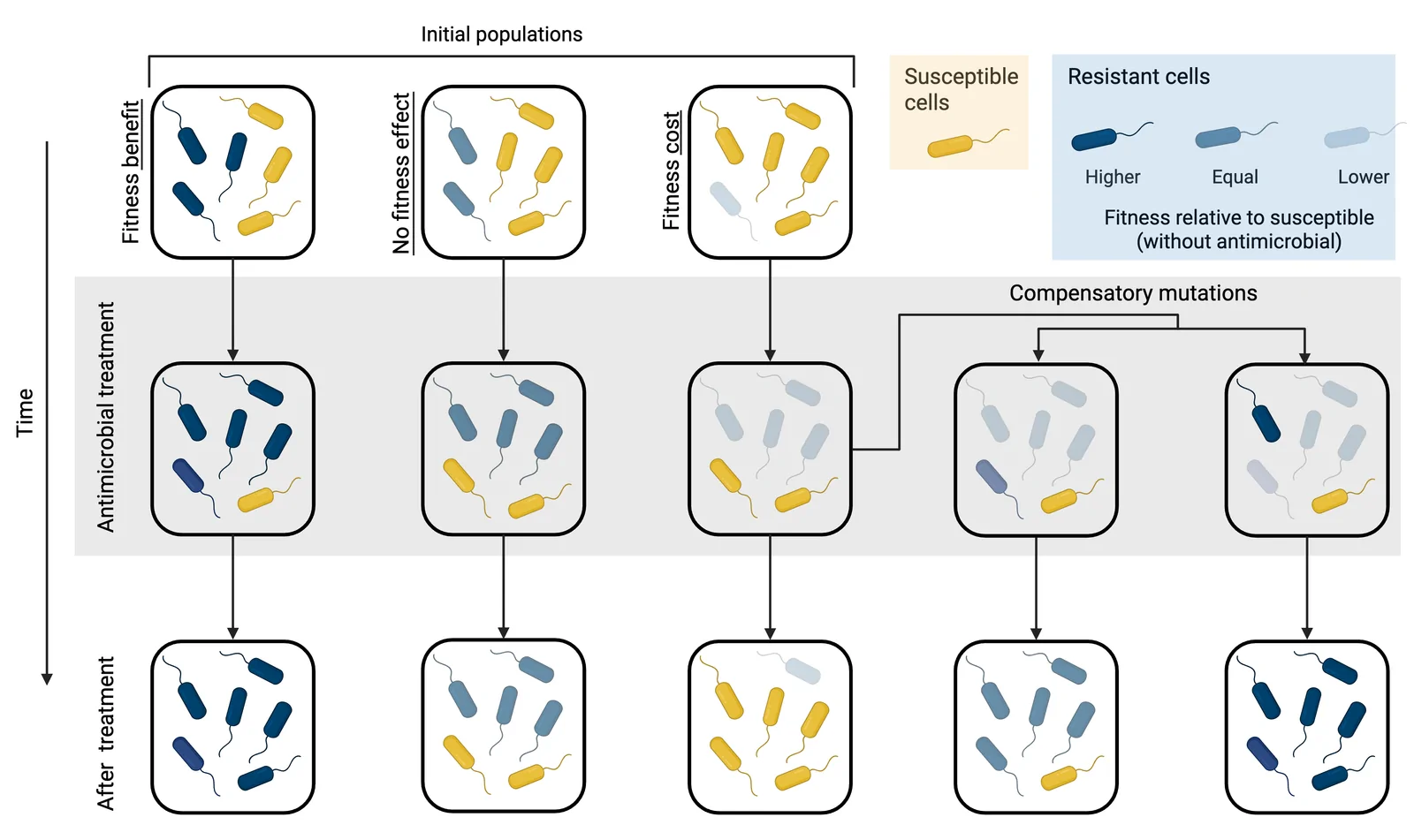
A schematic of the impact on fitness of AMR acquisition on bacteria following treatment with an antimicrobial. Cells which are resistant or susceptible to the antimicrobial may co-exist in the population. Resistant cells can have higher, equal or lower fitness than susceptible cells (top panels). Upon antimicrobial treatment, resistant cells are selected for and their frequency increases in the population. After treatment, if resistant cells have higher fitness than susceptible cells, they may replace the latter in the population. If fitness is equal, either or both types of cells may persist, while if resistant cells have a fitness defect, their frequency is likely to decrease in the population after antimicrobial treatment. Compensatory mutations can complicate the use of fitness as a collateral effect by alleviating the fitness cost of antimicrobial-resistant mutations, allowing the originally less fit resistant cells to persist or become dominant. Created with BioRender.com.

The fitness costs associated with AMR acquisition can be variable, and diverse factors influence the fitness effect of AMR acquisition, making fitness costs difficult to predict [48–50]. Fitness costs can vary in different strains due to *epistasis* [51, 52] (Table 1), which can include mutations acquired later in evolution, such as compensatory mutations [45, 53]. For example, the observed fitness effects of acquisition of pOXA-48 ranged from >20% fitness cost to an ~20% fitness benefit, which was influenced by the genetic background at both a species and strain level [52].

The environment in which the mutation or AMR gene is expressed can also impact fitness costs [54]. Environmental effects are of particular interest as many studies on AMR and fitness effects are performed in nutrient-rich laboratory growth media, which do not always translate to the infection-relevant environment, such as urine [48, 55]. Another important but often overlooked consideration is the range of mutations and AMR genes which can be acquired to confer resistance to a single antimicrobial (or antimicrobial class), which can result in diverse fitness effects [29].

Fitness costs can also be indirectly related to the acquisition of AMR itself. For example, they may result from the burden of replicating large plasmids [44, 56], deleterious genes or genetic variants within a plasmid or linked to the AMR gene or variant [43]. Furthermore, it is important to note that AMR does not always result in fitness costs in the absence of the antibiotic and, in some cases, can even result in fitness benefits [48, 49, 57] (Fig. 1).

Despite the challenges of characterizing the fitness cost associated with AMR acquisition, the idea that acquisition of AMR leads to a reduction of fitness in the absence of the antimicrobial has led to the implementation of strategies to reduce the incidence of AMR by removing antibiotics from use, with varying degrees of success [40, 58, 59]. For example, reducing the national clinical prescription rate of trimethoprim for 24 months did not reduce resistance in *E. coli* strains isolated from urinary tract infections (UTIs) in Kronoberg County, Sweden. Instead, a slight increase in the prevalence of trimethoprim resistance was identified [60]. In contrast, it was found that the reduction in quinolone prescriptions resulted in an immediate decrease in resistance in Israel, equating to a 1.16% reduction in quinolone-resistant *E. coli* per 1,000 defined daily doses [59]. Additionally, in Malawi, the reduction in use of chloramphenicol for sepsis coincided with an increase in the incidence of chloramphenicol-susceptible *E. coli* and *Klebsiella pneumoniae* [61, 62].

While strategies based on fitness costs have had mixed results, they still have the potential to succeed. However, robust, comprehensive *in vitro* studies which consider diverse mechanisms of resistance are needed to underpin these approaches, with the most impactful interventions prioritized for further study. In addition, other factors which influence fitness costs should be considered, such as the likelihood of the acquisition of compensatory mutations [63] and further factors that may impact the transmission dynamics of resistant pathogens (e.g. socioeconomic impact) [6].

## Collateral susceptibility

Acquisition of AMR mutations and genes which confer resistance to an antimicrobial from a particular class can lead to changes in susceptibility to antimicrobials from a different, unrelated class. Changes in antimicrobial susceptibility can come in two forms, an increase in susceptibility, termed *collateral susceptibility* (Table 1), or a decrease in susceptibility, termed *collateral resistance* (Table 1, Fig. 2). It has been proposed that such collateral effects can be exploited to design treatment regimens [31, 35, 64, 65]. For example, if the mutations or AMR genes that result in resistance to antimicrobial A lead to an increase in susceptibility to antimicrobial B, then antimicrobial B can be recommended for a patient whose infection has developed resistance to antimicrobial A, or as an alternative to antimicrobial A if resistance has become highly prevalent within the community or hospital setting. For example, 10 *E. coli* strains evolved *in vitro* to be ciprofloxacin resistant demonstrated conserved collateral susceptibility to gentamicin (8/10) and fosfomycin (7/10) [66]. Conserved collateral resistance to other antimicrobials, including chloramphenicol and temocillin, was also observed [66]. Imamovic *et al*. found that lab-adapted ciprofloxacin-resistant *Pseudomonas aeruginosa* with mutations in *nfxB* were associated with collateral susceptibility to some aminoglycosides and *β*-lactams [67]. Importantly, they demonstrated convergence with longitudinal clinical data from a cystic fibrosis patient, which showed clearance of subpopulations with *nfxB* mutations following aminoglycoside and *β*-lactam treatment [67].

**Fig. 2.**
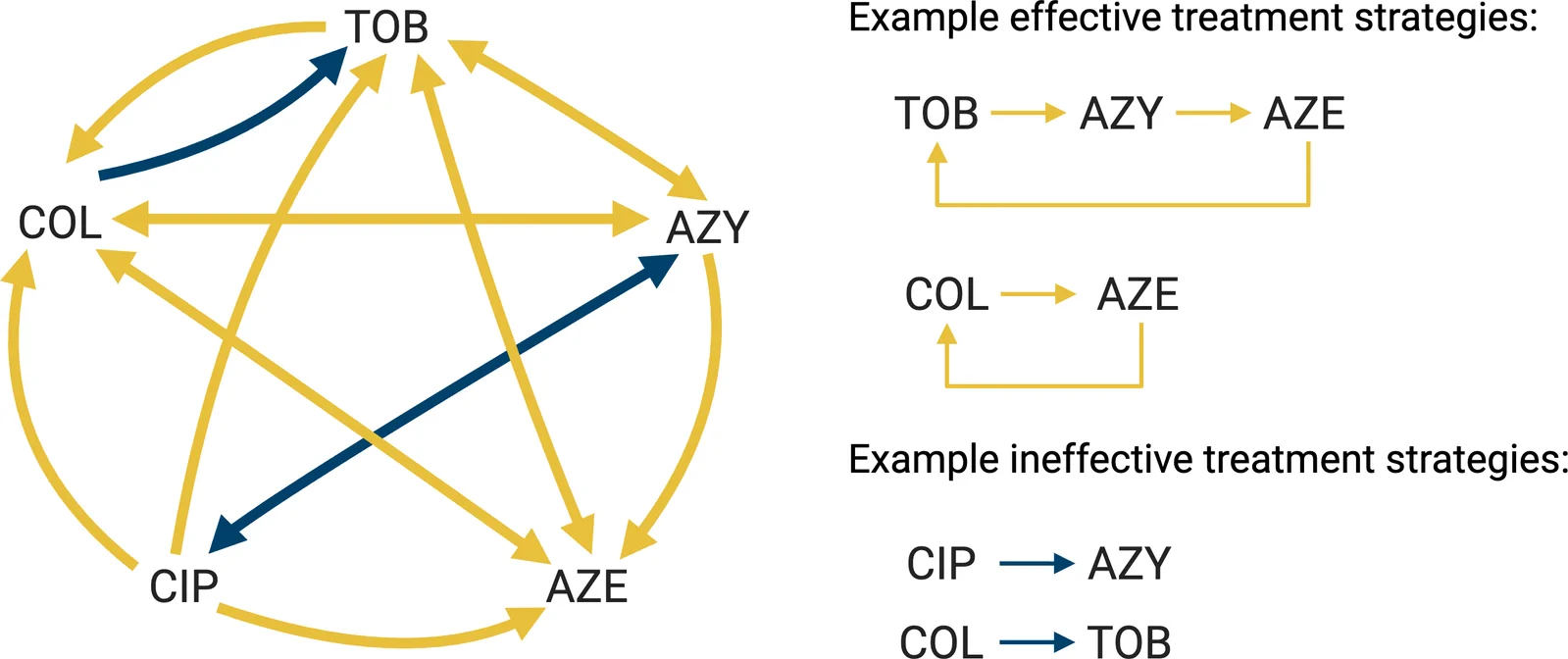
Example of a collateral susceptibility network from Imamovic *et al*. [67], where yellow lines indicate collateral susceptibility, blue lines indicate collateral resistance and no interconnecting lines indicate no effect. The direction of the arrows indicates the direction of collateral effect; e.g. resistance to colistin (COL) results in collateral resistance to tobramycin (TOB). Antibiotics depicted are tobramycin (TOB), azithromycin (AZY), aztreonam (AZE), ciprofloxacin (CIP) and colistin (COL). Created with BioRender.com.

In terms of prolonging the lifetime of antimicrobials, the ideal situation would be the identification and characterization of conserved networks of antimicrobials which display collateral susceptibility (Fig. 2). Therefore, if resistance arises to one antimicrobial, this would increase susceptibility to the other, reducing the potential for resistance development to both antimicrobials compared to antimicrobial pairs which do not show collateral susceptibility [68]. Collateral susceptibility networks have been studied *in vitro* and *in vivo* in a range of bacterial species against many antimicrobial agents [64, 68–71]. Results from these studies have shown the potential benefits of exploiting collateral networks through combination treatments of antimicrobials and antibiotic cycling [31, 64, 66–71]. The rationale behind antimicrobial cycling and combination is that switching treatment prevents the long-term prevalence of the resistant strains, as resistance is unstable or can be reversed. However, much of this work has only been performed *in vitro*.

Patterns of collateral effects have been investigated in experimentally evolved independent populations of *P. aeruginosa* PA14 against commonly used antimicrobials [69]. Some replicate populations that were adapted to cefsulodin were susceptible to gentamicin and streptomycin, whereas others showed neutral effects or collateral resistance to the same antimicrobials [69]. Additionally, a few replicate populations adapted to gentamicin displayed collateral susceptibility to piperacillin–tazobactam and carbenicillin, while the other replicate populations showed resistance against these antimicrobials [69]. The study demonstrated that antimicrobial selection of diverse mutational profiles can lead to different collateral susceptibility/resistance outcomes.

Another study [70] highlighted the potential of drug cycling to reduce AMR by investigating single, combined and alternating treatments of trimethoprim/sulfamethoxazole, ciprofloxacin and neomycin against *Staphylococcus aureus* and found that alternating treatments had the least likelihood of developing resistance due to alternating the spectrum of activity of the antimicrobials. A meta-analysis of published outcomes of drug cycling found that antimicrobial cycling had a varied effect on mortality in intensive care units, depending on the study, with overall no significant changes [71]. However, there was a significant decrease observed in the acquisition of resistance in medical intensive care units due to antimicrobial cycling [71].

Antimicrobial combination treatments reduce the potential for resistance to arise by targeting multiple different mechanisms simultaneously. As with drug cycling, the impact of antimicrobial combination therapy is also mixed. A recent meta-analysis showed that, overall, the acquisition of resistance was reduced when fewer antimicrobials were combined compared to a higher number, although this was dependent on the infection and pathogens targeted [72]. Another meta-analysis [73] found that, overall, combination therapy was associated with lower mortality and higher clinical success compared to monotherapy for carbapenem-resistant Gram-negative infections. Again, this was pathogen- and infection-dependent [73].

An alternative approach to combination therapy is to combine two or more pharmacophores in a single chemical structure through medicinal chemistry to form hybrid antimicrobials [74, 75]. Despite not being a true hybrid antimicrobial as it is a combination of a cephalosporin and a siderophore, which enhances uptake into the cell, cefiderocol is illustrative of the potential of hybrid antimicrobials. This compound is a combination of the cephalosporin pharmacophore, combined with chlorocatechol, a siderophore [74], and used for the treatment of complicated UTI [74]. Several other hybrid antimicrobials have entered clinical trials, such as rifamycin–nitroimidazole for *Helicobacter pylori* and vaginosis [76]. However, combining pharmacophores based on collateral susceptibility has not been extensively explored.

The mechanisms behind collateral changes in susceptibility are not entirely clear, and this is a subject of study. In *Staphylococcus*, it has been shown that mutations in *gyrA* that cause resistance to ciprofloxacin can result in collateral effects due to genome-wide changes in transcription [77]. Collateral susceptibility between tobramycin (aminoglycoside) and nalidixic acid (quinolone) has been identified to be due to mutations in *trkH* [68]. Mutations in *trkH* reduce uptake of tobramycin by proton-motive force, the mechanism of uptake for aminoglycosides, while also reducing efflux of nalidixic acid and tetracycline by AcrAB-TolC, which is also dependent on proton-motive force [68]. However, in general, collateral susceptibility networks have been explored for the acquisition of specific AMR mutations and/or genes. Mechanisms of resistance to many antimicrobials are a result of the acquisition of diverse genes and the development of mutations in a wide range of genes. This complicates a non-targeted approach when implementing into clinical practice, as these observed collateral susceptibility networks may not be conserved across the diverse mechanisms of resistance. Additionally, there has been limited exploration of collateral susceptibility networks, which have only been mapped for a limited range of bacterial isolates, species and antimicrobials. For those collateral susceptibility networks that have been explored, some of the antimicrobials used to treat multidrug-resistant infections, such as colistin, are avoided due to their toxicity when used long-term. While other antimicrobial pairs which have been assessed lack clinical translation, they are not used to treat the same infection.

## Mutant selection windows

An emerging area of interest for consideration as a collateral effect, which could be harnessed to optimize antimicrobial prescribing, are *mutant selection windows* (MSWs, Table 1). MSW may potentially be used to predict the probability of the enrichment of spontaneous mutations conferring resistance or reduced susceptibility to an antimicrobial at concentrations within the therapeutic window. The MSW is the antimicrobial concentration range where it is possible to select for single-step mutations, that is, a single mutation that leads to decreased susceptibility [78]. This window falls between the *MIC* (Table 1) and the *mutant prevention concentration* (MPC, Table 1, Fig. 3). The MIC defines the *in vitro* susceptibility of a bacterial isolate to a specific antimicrobial [79]. Experimentally, the MIC is measured as the lowest concentration of an antimicrobial that inhibits the visible growth of a bacterial population over a defined period of time (e.g. 18 h). In some cases, the MIC is the same concentration as the *minimum bactericidal concentration* (MBC, Table 1), the lowest concentration of the antimicrobial to cause cell death of the susceptible population. However, the MBC is often higher than the MIC, and therefore, at concentrations above the MIC, bacterial cells may survive, at least for a period of time [80–82]. In the context of MSWs, the MIC is the lowest concentration of an antimicrobial which inhibits the growth of susceptible cells [78] (Fig. 3a). Mutations arise spontaneously and those which confer resistance or decreased susceptibility to an antimicrobial can be selected for below the MIC in the presence of the antimicrobial due to the competitive advantage over the susceptible population [78, 83–85]. The resistant population with mutations which confer resistance or decreased susceptibility will then expand, leading to the accumulation of mutations, which can contribute to decreased susceptibility or resistance to the antimicrobial [78]. Importantly, at sub-MIC concentrations, the susceptible population can still potentially continue to grow alongside the resistant/less susceptible population. However, at concentrations above the MIC, it is expected that these spontaneous mutations, which confer decreased susceptibility or resistance to the antimicrobial, are enriched for due to the antimicrobial inhibiting the growth of the susceptible population [78, 86–88]. Therefore, at concentrations above the MIC, the dominant population, or only population, will be those with spontaneous mutations which decrease susceptibility or confer resistance to the antimicrobial. The MPC was first described as a novel measurement of antimicrobial susceptibility [88], and traditionally, the MPC is measured using a much higher inoculum (≥10^9^ c.f.u. ml^−1^) than an MIC assay (~10^5^ c.f.u. ml^−1^) [87]. It was observed that at higher inoculums, subpopulations could arise which confer reduced susceptibility or resistance to the antimicrobial, usually through mutations [87, 88]. Therefore, the MPC is defined as the lowest antimicrobial concentration *in vitr*o which inhibits the growth of these subpopulations [87]. Podnecky *et al*. demonstrated that following selection with ciprofloxacin, the MPC to other antimicrobials (e.g. chloramphenicol, amoxicillin and gentamicin) can increase or decrease [66]. The increase or decrease in MSW led to a shift in the MSW and/or narrowing or widening of the MSW, theoretically affecting the concentration of the antimicrobial that can select for resistance or reduced susceptibility [66]. The acquisition of resistance to an antimicrobial can influence the potential for further selection of resistance mutations to the same or to other antimicrobials, as a result of epistatic interactions that impact the diversity and phenotypic effects of subsequent mutations that may arise. One way to measure these changes in epistatic interactions is to compare the MSWs of susceptible and resistant strains towards alternative antimicrobials. Therefore, MSWs have been suggested as a way to predict how an AMR strain may evolve further resistance to a second antimicrobial [78]. If the acquired resistance, either by mutations or acquisition of an antimicrobial-resistant gene, widens the MSW to a second antimicrobial, this second antimicrobial should be avoided, as it increases the ability of the strain to acquire further resistance. In contrast, narrowing the MSW for a second antimicrobial will decrease the ability to evolve resistance by mutation to the second antimicrobial and therefore, would be an attractive alternative.

**Fig. 3.**
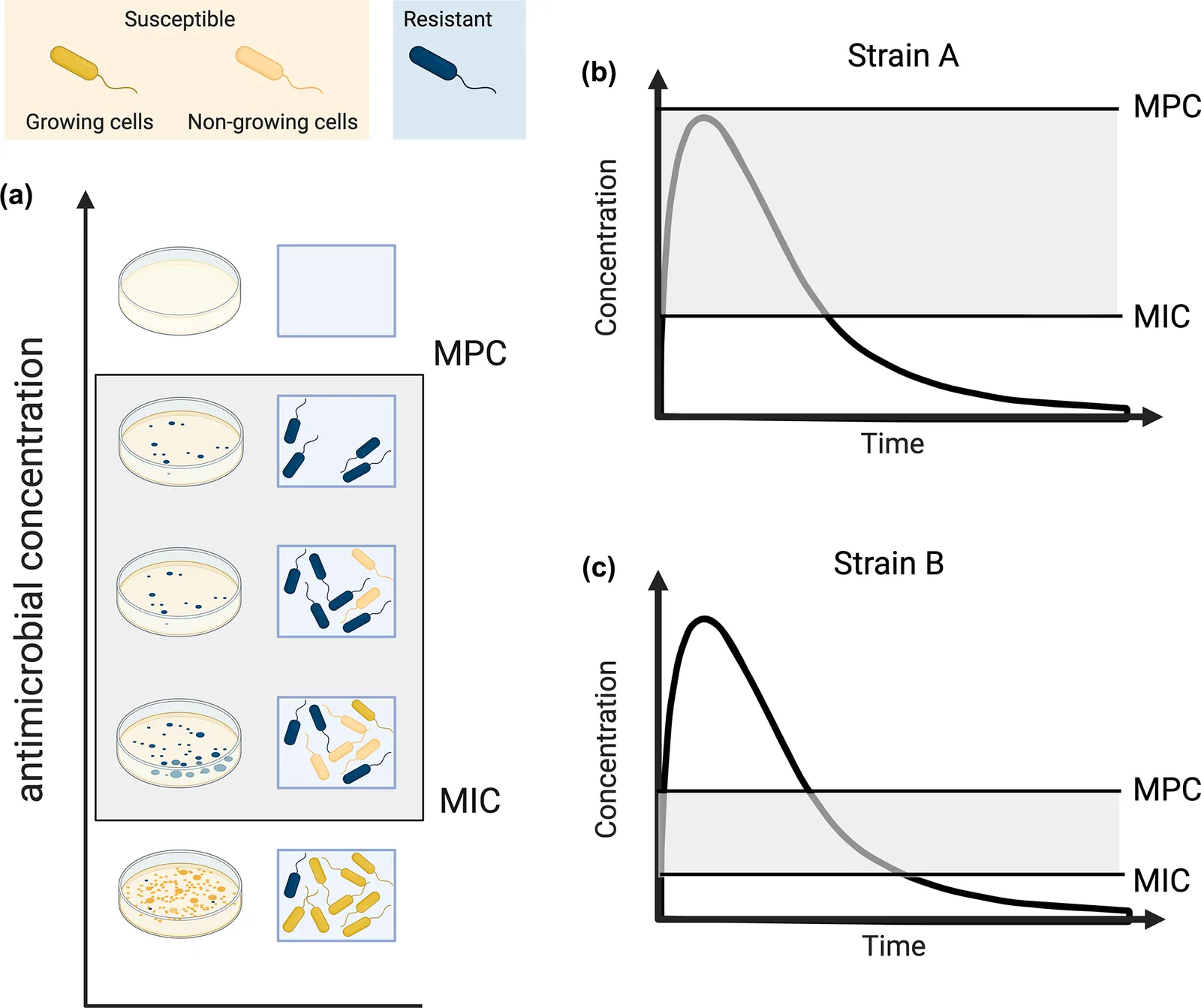
(a) Theoretical representation of the MSW: the window is defined as being between the MIC; above it is expected that spontaneous mutations which confer decreased susceptibility or resistance to the antimicrobial are enriched for while inhibiting the growth of the susceptible population, and the MPC, the lowest antimicrobial concentration that inhibits the growth of subpopulations which are resistant or have decreased susceptibility to the antimicrobial. Antimicrobial concentrations below the MIC will select for spontaneous mutations which confer resistance or decreased susceptibility due to competitive advantage, while the susceptible population can still potentially grow. Above the MPC, the antimicrobial concentration is too high for the subpopulations with resistance or decreased susceptibility to the antimicrobial to grow. Within this window, some susceptible cells may be able to survive, but their growth is mostly inhibited; as the antimicrobial concentration increases, they will be killed. Instead, those cells with decreased susceptibility or resistance will survive and grow. (**b, c**) Representations of the theory behind using MSW as a collateral effect, with the MSW overlaid over the pharmacokinetic/pharmacodynamic model of an antimicrobial concentration over time. Here, strain B is a derivative of strain A with resistance to antimicrobial 1. In (b), the MSW of strain A for antimicrobial 2 overlaps the peak antimicrobial concentration, suggesting that spontaneous mutants which have reduced susceptibility or are resistant will be selected for. However, in (c), the MSW of strain B is lower and therefore the concentration of antimicrobial 2 remains above the MPC for a long period of time, reducing the probability of selecting for spontaneous mutations which are resistant or have reduced susceptibility. Created with BioRender.com.

MSWs as a collateral effect have increasingly gained support and have been explored in a range of *in vitro* and *in vivo* studies, highlighting the potential use of MSW to mitigate AMR and to design drug pairs to limit the evolution of resistance [89–92]. James *et al*. identified that selection of resistance to trimethoprim resulted in a reduced MSW to nitrofurantoin in uropathogenic *E. coli* while having no effect on fosfomycin [93]. Also in *E. coli*, prior ciprofloxacin resistance increased the MSW to several antimicrobials, including chloramphenicol and amoxicillin and decreased the MSW to others, such as ertapenem and fosfomycin [66].

As with collateral susceptibility, assessment of the impact of AMR on MSWs has been relatively limited and confined to distinct antimicrobial pairs, limited bacterial strains and species and specific antimicrobial-resistant mutations or genes. Limited exploration of MSWs has so far constrained the clinical utility of this approach, and therefore, deeper and more extensive research on MSW is required to support implementation. Although controversial, there is a growing interest in using therapeutic drug monitoring to have tailored targets of free drug above the MIC to improve treatment success [78, 94–96]. A shift in the MSW due to collateral effects of acquisition of AMR to one antimicrobial may alter the length of time the antimicrobial concentration at the infection site is within the MSW for a second antimicrobial (Fig. 3b). The longer the therapeutic drug concentration is in the MSW, the higher the probability there is to enrich for resistance to the second antimicrobial. Therefore, robust pharmacokinetics/pharmacodynamics or therapeutic drug monitoring data is required to compare to determine if the shift in MSW impacts leads to the concentration of the antimicrobial at the infection site being below the MPC for a long period of time. Additionally, it would be important to standardize the MSW assay to assess against the minimum and maximum population size found in the specific infection site to ensure accuracy and robustness of the data.

## Relative population establishment probability

A resistant bacterial population must arise from at least a single bacterial cell that acquires resistance-conferring mutations or genes. This single cell may replicate, producing a resistant lineage, or may die before successfully replicating, therefore preventing the development of a resistant lineage (Fig. 4). The inability of a single resistant cell to successfully establish a population could be due to several factors. In the absence of antimicrobials, the fitness costs associated with carrying a resistance-conferring mutation or AMR gene might prevent the outgrowth of the resistant strain. In addition, as shown by Alexander and MacLean [97], the probability of outgrowth of a single resistant cell can be significantly reduced in the presence of sub-MIC levels of an antimicrobial, compared to its absence, likely due to the toxic effect of antimicrobials. Moreover, if the resistant cells have any fitness benefit in the presence of antimicrobials, the establishment of the population can still not be guaranteed due to the random and unpredictable nature of cell viability on a single-cell level.

**Fig. 4.**
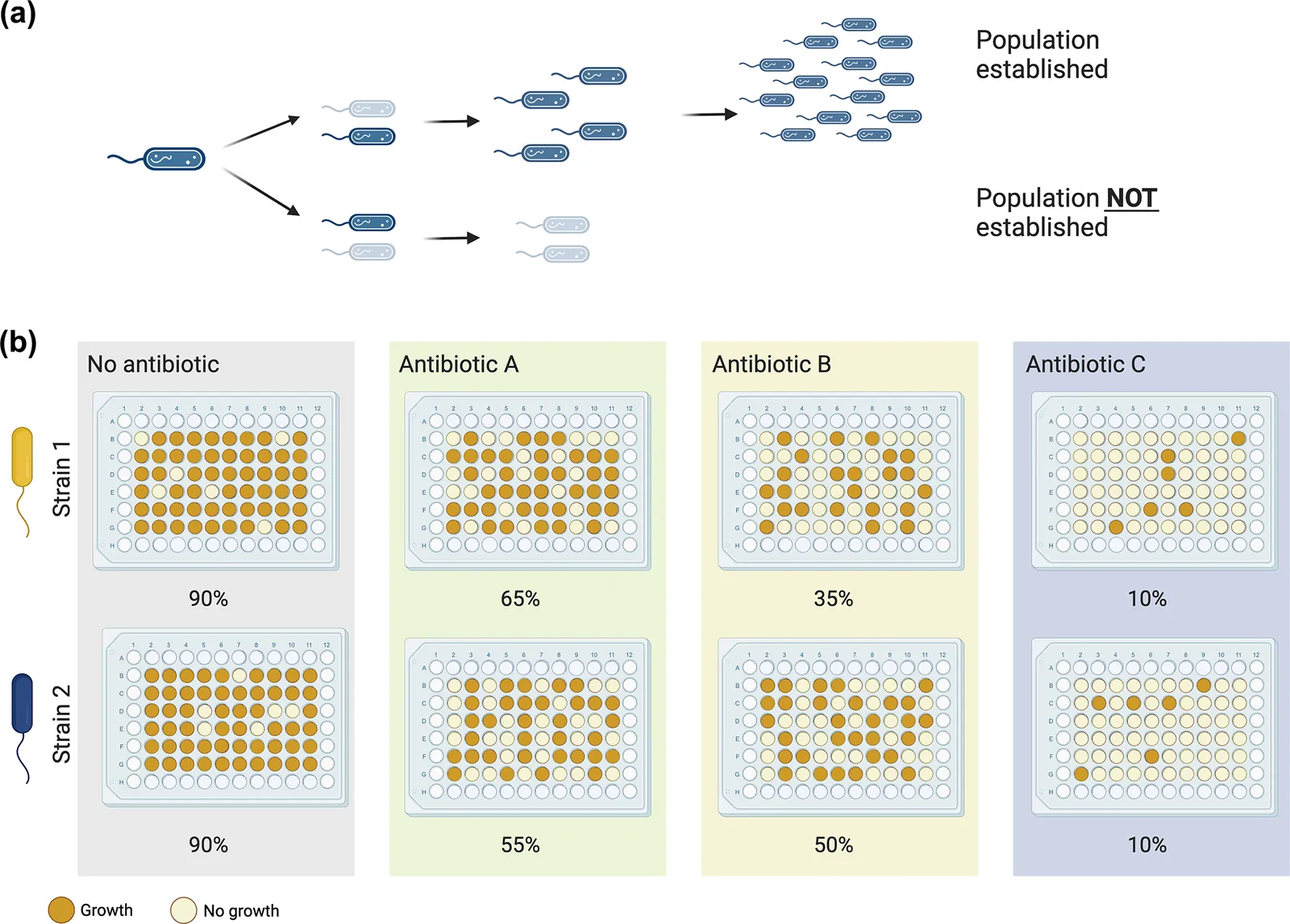
(a) A diagram of the concept of population establishment probability. A single cell has two potential paths, one where it successfully replicates and is consequently able to establish a population. In the second, the cell dies before being able to replicate, resulting in the population being unable to establish itself. Relative population establishment probability may vary depending on the acquisition of AMR and the environment the cell is in. (**b**) This is a visual representation of how relative population establishment probability can be used as a collateral effect. It is important to note that due to the Poisson distribution, where some wells would contain no cells and others contain more than one cell, the number of wells with growth would not directly equal probability. The acquisition of AMR could increase or decrease the relative population establishment probability compared to the susceptible cell. If the acquisition of resistance can decrease the probability that a population can be established, it may be harnessed to limit the spread of AMR. Alternatively, the acquisition of AMR may vary when in the presence of two different antimicrobials to which the cell is susceptible. Therefore, it may be possible to select a second antimicrobial, for example, antibiotic C, which reduces the probability of a population being established when resistance arises to the first antimicrobial. Created with BioRender.com.

Relative *population establishment probability* (Table 1) is, therefore, another emerging area to be considered as a collateral effect. Relative population establishment probability enables the evaluation of the effect of AMR acquisition on the ability of bacteria to establish a population from a single cell relative to the absence of the AMR gene or mutation [97]. Although the number of cells per well within the assay follows a Poisson distribution, where some wells would contain no cells and others contain more than one cell, differences can be distinguished between strains and conditions (e.g. presence of different antimicrobials). Therefore, by measuring relative differences in population establishment probability (Fig. 4b) rather than absolute probability, it may be possible to apply the concept of relative population establishment probability to predict if the acquisition of AMR will increase or decrease population establishment probability relative to the same strain without the AMR gene or mutation. For example, Alexander and MacLean [97] found that the probability of establishment of a resistant strain is <5% if streptomycin is used at a concentration of 1/8 the value of the MIC in *P. aeruginosa*. Furthermore, knowledge of the differences in population establishment probability can be used to assess whether AMR acquisition conferring resistance to one antimicrobial can affect the relative population establishment probability in the presence of a second antimicrobial. James *et al*. developed trimethoprim-resistant derivatives of *E. coli* isolated from UTIs and tested the population establishment probability of these derivatives to two second-line UTI antimicrobials [93]. They found that trimethoprim resistance in UTI-isolated *E. coli* led to no significant change in population establishment in the presence of nitrofurantoin or fosfomycin relative to their trimethoprim-susceptible ancestors. However, this is only one example, and differences in establishment probabilities may still occur. Relative to fitness and collateral susceptibility, population establishment probability is a very new concept and lacks the required depth of exploration. To date, it has been insufficiently studied across diverse species, strains and antimicrobials, and the impact of the environment and microbiome needs to be investigated to be fully realized as a collateral effect. The microbiome is of particular interest when considering patients who have been colonized, especially with multidrug-resistant bacteria. Therefore, as with MSW, there needs to be large-scale studies to fully explore and interrogate these collateral effects.

## Barriers to translation

Until now, we have summarized the collateral effects which can be exploited to optimize new and existing antimicrobials to limit the impact of AMR. However, factors such as different genetic background of bacterial strains and the limitations of replicating the infection environment *in vitro* can affect the translation of collateral effects into clinics and limit their success.

## Genetic background

The collateral response of AMR-conferring mutations or genes may vary depending on the genome in which they occur due to genetic interactions (*epistasis*; Table 1) between the gene variants that confer AMR and other variants present in the genome. Epistasis is prevalent in biological networks and significantly affects the relationship between individual genotypes and phenotypes [98] and can therefore influence all the collateral effects we have described.

The fitness cost of a particular mutation may not be identical in diverse strains of the same species, as demonstrated for fluoroquinolone resistance mutations in *Campylobacter jejuni* [99], where a mutation, leading to the C257T amino acid change, in *gyrA* resulted in a fitness cost in one genetic background (62101S1) and a fitness gain on another (62301S2). Furthermore, genetic background can also influence the resistance-conferring mutations that arise during adaptation to antimicrobials. An *E. coli* strain that evolved resistance to amoxicillin developed mutations in the *ampC* promoter (*β*-lactamase), *ompR* (transcriptional regulator), *citF* (citrate lyase) and *rpoD* (Sigma 70). However, when the same *E. coli* strain containing a mutation conferring resistance to enrofloxacin was evolved to amoxicillin resistance, an additional mutation in *rpoA* developed [100]. As such, mutations that appear during the development of resistance to a second antimicrobial are not always the same mutations that will appear against that specific antimicrobial in a different or altered background. From these studies, it can be concluded that epistasis plays an important role in the acquisition of AMR as well and may complicate the outcomes of evolutionary studies. It is therefore imperative that studies explore a wide range of different species, strains and sequence types to fully understand the collateral effects of AMR.

## Polymicrobial environments

Many of the above collateral effects are assessed in single-strain, monoculture environments. However, the fact is often overlooked that in patients, infections can occur in the presence of microbial communities and/or be polymicrobial, which consist of multiple bacterial pathogens. In polymicrobial infections, inter- and intra-species interactions may, therefore, alter the response of bacteria to antimicrobials [101] (Fig. 5). For example, in a community of two species, *Salmonella enterica* serovar Typhimurium was able to survive a lethal concentration of ampicillin due to enzymatic inactivation of the drug by *β*-lactamase-producing *E. coli* under laboratory conditions [102]. Another study on the effect of multispecies interactions on antimicrobial susceptibility has found that the survival of chloramphenicol-susceptible *Streptococcus pneumoniae* increased in the presence of a chloramphenicol-inactivating acetyltransferase-producing resistant strain of *S. aureus* [103]. In addition to altering the response to antimicrobials, polymicrobial networks also affect the evolution of AMR in bacteria. For example, resistance evolved more rapidly in patients infected with a mixed population of *P. aeruginosa* than in those colonized with a single susceptible strain [104]. However, this study was limited to 13 patients and therefore requires detailed further investigation. Meanwhile, the presence of a member of the microbial community either increased or had no detrimental effect on conjugation efficiency in uropathogenic *E. coli* [105], which could influence the probability of acquiring AMR plasmids. Finally, the acquisition of AMR has been shown to influence the ability of pathogens, including *S. aureus* and *P. aeruginosa*, to invade into an *in vitro* constructed single- and multi-species community. However, this effect is dependent on the composition of the community and species used [106, 107]. Taken together, these findings demonstrate the importance of assessing the acquisition of AMR and the collateral effects of AMR in polymicrobial communities. While it is important to build scientific knowledge from simplistic models to more complex ones, these studies highlight the need for more realistic environments to improve clinical translation of AMR studies. The role of the microbiome in AMR is explored further in the perspective by Pittaccio *et al*. [108].

**Fig. 5.**
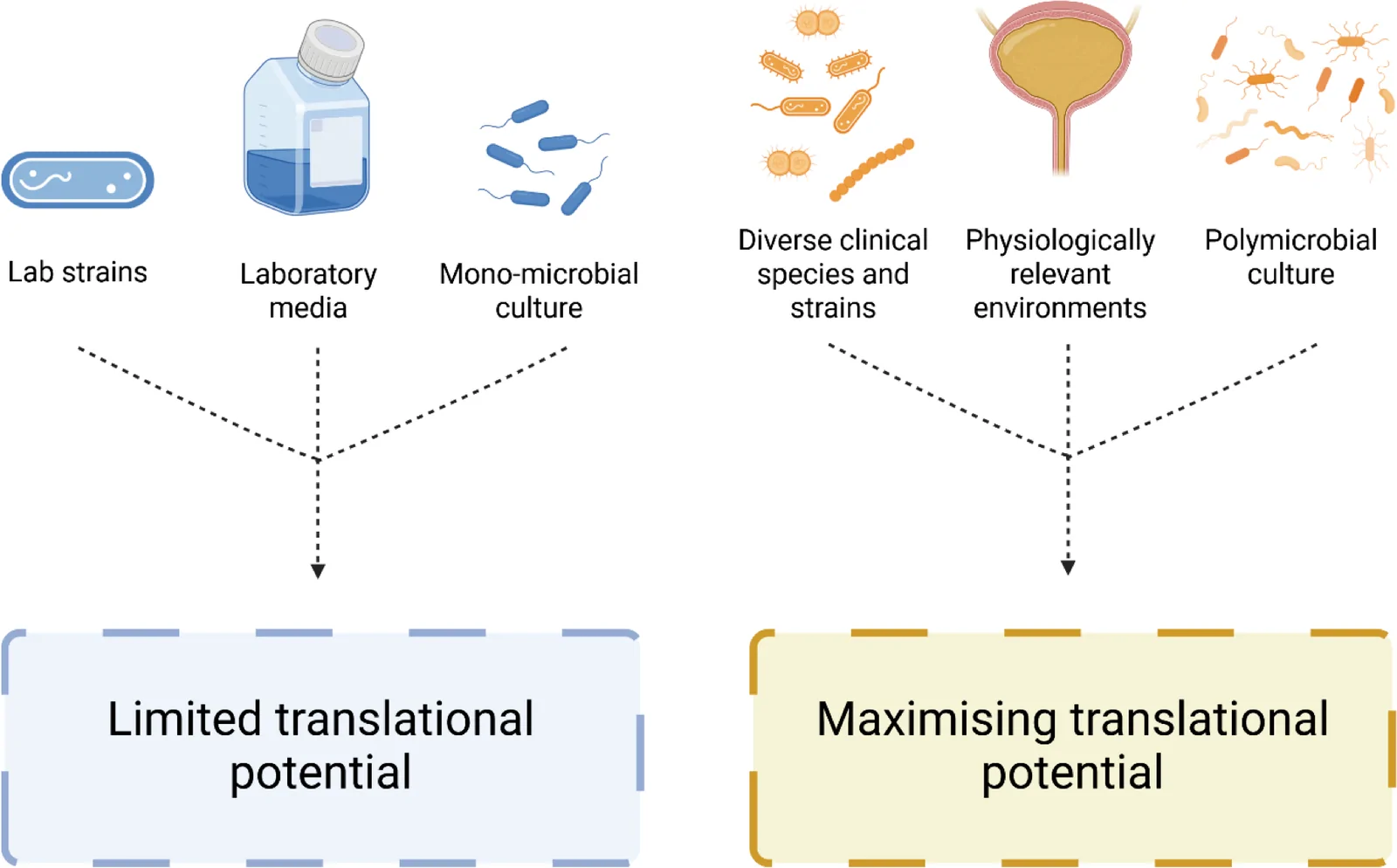
Schematic showing the barriers to maximizing potential (left) and the proposed changes required to increase and maximize translational potential for studying AMR and collateral effects (right). Created with BioRender.com.

## Physiologically relevant environments

One of the major barriers to the translation of *in vitro* studies on collateral effects to the clinic is the mismatch between the *in vitro* and *in vivo* environments. The majority of the studies we have highlighted here have been performed in laboratory media, often nutrient-rich, which is not reflective of the infection environment. Even media that have been developed to match the *in vivo* environment can lack key metabolites produced during infection [109]. Furthermore, there are several key factors within hosts which have been demonstrated *in vitro* to potentially influence AMR acquisition, including inflammation [110], temperature [111] and the microbiome [105]. Infection-relevant conditions are often difficult to replicate *in vitro* and are therefore regularly overlooked when assessing microbial evolution.

The rationale for standardization in antimicrobial susceptibility testing (AST) in clinical microbiology and early-stage drug development, both nationally and internationally, is clear. However, many studies have highlighted the importance of the environment in which bacteria are in on the assessment of AST [55], fitness [55], biofilms [112] and selection of resistance [55, 113], among others. This is equally true when considering more physiologically relevant environments [48, 114]. Animal models have been used previously, although they are often biologically divergent from humans. An excellent example of divergence between animal models and humans is the difference in structure and function of the murine bladder, often used to study UTIs, compared to the human bladder and demonstrating a need for models which more closely mimic the human bladder [115]. Cell culture models exist which closely mimic specific human infection sites and have been developed primarily to study host-pathogen interaction [116, 117]. However, the use of cell culture models which mimic the infection environment in studying AMR, despite growing interest in using more physiologically relevant environments, and their implementation to investigate collateral effects are, to our knowledge, limited [67, 118]. There is also a growing need to optimize cell culture models from a pathogen response standpoint and utilize these in AMR studies and the drug development pipeline to improve translation.

It is important to note that many studies on AMR evolution and bacterial responses to antimicrobials are often focused only on specific bacteria-antimicrobial combinations. These studies often do not consider the role of other unrelated, non-antimicrobial therapeutics a patient is on. A recent study [119] has shown the impact of non-antimicrobials have on AMR development in *E. coli*. Here, the authors found that mutation frequency in an *E. coli* isolate increased when ciprofloxacin was combined with either ibuprofen or acetaminophen relative to ciprofloxacin alone [119]. As such, non-antimicrobial therapeutics may affect the activity of an antimicrobial, the acquisition of AMR and the consequence of collateral effects. Therefore, drug–drug interaction is another important aspect to account for when mimicking real-world conditions.

## Conclusion

The rise of AMR poses a significant threat to global healthcare and the economy, highlighting the urgency for effective strategies to address AMR. Understanding the role of collateral effects of AMR is important for preserving the efficacy of existing antibiotics and improving treatment outcomes. Several key collateral effects have been discussed in this perspective: the fitness costs of AMR acquisition, collateral susceptibility, MSW and population establishment probability. We also highlighted the importance of considering epistasis, polymicrobial infections and the effect of the environment on the assessment of collateral effects. These principles offer invaluable insights into the evolution of AMR and its potential impact on treatment outcomes.

However, collateral effects are often assessed separately, without consideration for other potentially linked collateral effects. Therefore, it is important to take a holistic approach to assess collateral effects to provide a comprehensive picture of the phenotypic effect of AMR and how collateral effects could be harnessed to optimize antimicrobial prescribing. In addition, many studies are performed in mono-microbial conditions, using nutrient-rich laboratory media and laboratory bacterial strains and/or limited species or strain diversity. This does not accurately reflect the complex infection environment and the diversity of pathogenic bacteria and therefore limits translation (Fig. 5).

To effectively combat AMR, it is necessary to continue research efforts that not only study collateral effects but also consider their translation into clinical applications. Several strategies have been applied to reduce AMR, including antimicrobial stewardship, with varying success, and the WHO, and NAPs have again highlighted the need to optimize antimicrobial prescribing. It is therefore important to invest in the exploration of alternative strategies, based on robust *in vitro* data, such as collateral effects. Additionally, *in vitro* assessment of collateral effects could be embedded within the lead optimization stage of the drug development pipeline. Here, tweaks can be made to the antimicrobial to reduce the potential of resistance development and assess the impact of AMR on other already marketed antimicrobials and new antimicrobials. Detailed *in vitro* data on antimicrobials could inform the preclinical and clinical phases on the optimal use to limit AMR emergence and maintain their efficacy prior to market entry. Harnessing collateral effects to optimize antimicrobial therapy will add another strategy to our arsenal to mitigate the impact of AMR on global health.

## Abbreviations

AMR: antimicrobial resistance
AST: antimicrobial susceptibility testing
HGT: horizontal gene transfer
MBC: minimum bactericidal concentration
MGE: mobile genetic element
MPC: mutant prevention concentration
MSW: mutant selection window
NAPs: national action plans
UTI: urinary tract infection
WHO: World Health Organization

## References

[1] Dadgostar P (2019). Antimicrobial resistance: implications and costs. Infect Drug Resist.

[2] Prestinaci F, Pezzotti P, Pantosti A (2015). Antimicrobial resistance: a global multifaceted phenomenon. Pathog Glob Health.

[3] Naghavi M, Vollset SE, Ikuta KS, Swetschinski LR, Gray AP (2024). Global burden of bacterial antimicrobial resistance 1990–2021: a systematic analysis with forecasts to 2050. The Lancet.

[4] Cooper LN, Beauchamp AM, Ingle TA, Diaz MI, Wakene AD (2024). Socioeconomic disparities and the prevalence of antimicrobial resistance. Clin Infect Dis.

[5] Ayorinde A, Ghosh I, Ali I, Zahair I, Olarewaju O (2023). Health inequalities in infectious diseases: a systematic overview of reviews. BMJ Open.

[6] El-Maradny YA, Nortey MA, Hakayuwa CM, Anyamene EL, Mary JJF (2025). The impact of socioeconomic disparities climate factors and antimicrobial stewardship on antimicrobial resistance in Africa. Discov Public Health.

[7] Ljungqvist G, van Kessel R, Mossialos E, Saint V, Schmidt J (2025). Mapping socioeconomic factors driving antimicrobial resistance in humans: an umbrella review. One Health.

[8] Reygaert WC (2018). An overview of the antimicrobial resistance mechanisms of bacteria. AIMS Microbiol.

[9] Aslam B, Wang W, Arshad MI, Khurshid M, Muzammil S (2018). Antibiotic resistance: a rundown of a global crisis. Infect Drug Resist.

[10] Ho CS, Wong CTH, Aung TT, Lakshminarayanan R, Mehta JS (2025). Antimicrobial resistance: a concise update. Lancet Microbe.

[11] Kumar P, Gulati P, Thakur M, Kamal R (2026). Global strategies for combating antimicrobial resistance. Infect Disord Drug Targets.

[12] Bo L, Sun H, Li Y-D, Zhu J, Wurpel JND (2024). Combating antimicrobial resistance: the silent war. Front Pharmacol.

[13] Ahmed SK, Hussein S, Qurbani K, Ibrahim RH, Fareeq A (2024). Antimicrobial resistance: Impacts, challenges, and future prospects. J Med Surg Public Health.

[14] Fraser AJ, Wilkinson MC, Meehan CJ, Casewell NR, Roberts AP (2026). Screening the antimicrobial properties of snake venoms: an iterative fractionation approach. Access Microbiol.

[15] Roberts AP (2020). Swab and Send: a citizen science, antibiotic discovery project. Future Sci OA.

[16] Theuretzbacher U, Outterson K, Engel A, Karlén A (2020). The global preclinical antibacterial pipeline. Nat Rev Microbiol.

[17] Butler MS, Henderson IR, Capon RJ, Blaskovich MAT (2023). Antibiotics in the clinical pipeline as of December 2022. J Antibiot.

[18] Antoniou V, Somani BK (2022). Topical and oral oestrogen for recurrent urinary tract infection-evidence-based review of literature, treatment recommendations, and correlation with the European association of urology guidelines on urological infections. Eur Urol Focus.

[19] Yadegar A, Bar-Yoseph H, Monaghan TM, Pakpour S, Severino A (2024). Fecal microbiota transplantation: current challenges and future landscapes. Clin Microbiol Rev.

[20] Strathdee SA, Hatfull GF, Mutalik VK, Schooley RT (2023). Phage therapy: From biological mechanisms to future directions. Cell.

[21] Zay Ya K, Win PTN, Bielicki J, Lambiris M, Fink G (2023). Association between antimicrobial stewardship programs and antibiotic use globally: a systematic review and meta-analysis. JAMA Netw Open.

[22] Mulchandani R, Tiseo K, Nandi A, Klein E, Gandra S (2025). Global trends in inappropriate use of antibiotics, 2000-2021: scoping review and prevalence estimates. BMJ Public Health.

[23] Shanmugakani RK, Srinivasan B, Glesby MJ, Westblade LF, Cárdenas WB (2020). Current state of the art in rapid diagnostics for antimicrobial resistance. Lab Chip.

[24] Peri AM, Chatfield MD, Ling W, Furuya-Kanamori L, Harris PNA (2024). Rapid diagnostic tests and antimicrobial stewardship programs for the management of bloodstream infection: what is their relative contribution to improving clinical outcomes? A systematic review and network meta-analysis. Clin Infect Dis.

[25] Strathdee SA, Davies SC, Marcelin JR (2020). Confronting antimicrobial resistance beyond the COVID-19 pandemic and the 2020 US election. The Lancet.

[26] World Health Organization Library of AMR national action plans. https://www.who.int/teams/surveillance-prevention-control-AMR/national-action-plan-monitoring-evaluation/library-of-national-action-plans.

[27] World Health Organization WHO’s contribution towards health outcomes. https://www.who.int/about/accountability/results/who-results-report-2022-mtr/outcome.

[28] Patel J, Moghaddam SS, Ranganathan S, Vezeau N, O’Neill E (2026). Global policy responses to antimicrobial resistance, 2021-22: a systematic governance analysis of 161 countries and territories. Lancet Infect Dis.

[29] Chavada J, Muneshwar KN, Ghulaxe Y, Wani M, Sarda PP (2023). Antibiotic resistance: challenges and strategies in combating infections. Cureus.

[30] Laborda P, Gil-Gil T, Martínez JL, Hernando-Amado S (2024). Preserving the efficacy of antibiotics to tackle antibiotic resistance. Microb Biotechnol.

[31] Roemhild R, Andersson DI (2021). Mechanisms and therapeutic potential of collateral sensitivity to antibiotics. PLoS Pathog.

[32] Mahmud HA, Wakeman CA (2024). Navigating collateral sensitivity: insights into the mechanisms and applications of antibiotic resistance trade-offs. Front Microbiol.

[33] Maeda T, Furusawa C (2024). Laboratory evolution of antimicrobial resistance in bacteria to develop rational treatment strategies. Antibiotics.

[34] Holguín H, Amariles P, Ospina W (2017). Interacciones evolutivas como un posible mecanismo de interacción medicamentosa: una aproximación para el control de la resistencia bacteriana. Rev chil infectol.

[35] Pál C, Papp B, Lázár V (2015). Collateral sensitivity of antibiotic-resistant microbes. Trends Microbiol.

[36] Orr HA (2009). Fitness and its role in evolutionary genetics. Nat Rev Genet.

[37] Andersson DI (2006). The biological cost of mutational antibiotic resistance: any practical conclusions?. Curr Opin Microbiol.

[38] Andersson DI, Hughes D (2010). Antibiotic resistance and its cost: is it possible to reverse resistance?. Nat Rev Microbiol.

[39] Melnyk AH, Wong A, Kassen R (2015). The fitness costs of antibiotic resistance mutations. Evol Appl.

[40] Holberger LE, Hayes CS (2009). Ribosomal protein S12 and aminoglycoside antibiotics modulate A-site mRNA cleavage and transfer-messenger RNA activity in *Escherichia coli*. J Biol Chem.

[41] Vanacker M, Lenuzza N, Rasigade J-P (2023). The fitness cost of horizontally transferred and mutational antimicrobial resistance in *Escherichia coli*. Front Microbiol.

[42] San Millan A, MacLean RC (2017). Fitness costs of plasmids: a limit to plasmid transmission. Microbiol Spectr.

[43] Rajer F, Sandegren L (2022). The role of antibiotic resistance genes in the fitness cost of multiresistance plasmids. *mBio*.

[44] Hall JPJ, Wright RCT, Harrison E, Muddiman KJ, Wood AJ (2021). Plasmid fitness costs are caused by specific genetic conflicts enabling resolution by compensatory mutation. PLoS Biol.

[45] de Vos M, Müller B, Borrell S, Black PA, van Helden PD (2013). Putative compensatory mutations in the rpoC gene of rifampin-resistant *Mycobacterium tuberculosis* are associated with ongoing transmission. Antimicrob Agents Chemother.

[46] Andersson DI, Levin BR (1999). The biological cost of antibiotic resistance. Curr Opin Microbiol.

[47] San Millan A (2018). Evolution of Plasmid-Mediated Antibiotic Resistance in the Clinical Context. Trends Microbiol.

[48] Hinz A, Amado A, Kassen R, Bank C, Wong A (2024). Unpredictability of the fitness effects of antimicrobial resistance mutations across environments in *Escherichia coli*. Mol Biol Evol.

[49] Vogwill T, MacLean RC (2015). The genetic basis of the fitness costs of antimicrobial resistance: a meta-analysis approach. Evol Appl.

[50] Alonso-Del Valle A, León-Sampedro R, Rodríguez-Beltrán J, DelaFuente J, Hernández-García M (2021). Variability of plasmid fitness effects contributes to plasmid persistence in bacterial communities. Nat Commun.

[51] Moura de Sousa J, Balbontín R, Durão P, Gordo I (2017). Multidrug-resistant bacteria compensate for the epistasis between resistances. PLoS Biol.

[52] Fernández-Calvet A, Toribio-Celestino L, Alonso-Del Valle A, Sastre-Dominguez J, Valdes-Chiara P (2023). The distribution of fitness effects of plasmid pOXA-48 in clinical enterobacteria. Microbiology.

[53] Goig GA, Menardo F, Salaam-Dreyer Z, Dippenaar A, Streicher EM (2023). Effect of compensatory evolution in the emergence and transmission of rifampicin-resistant *Mycobacterium tuberculosis* in Cape Town, South Africa: a genomic epidemiology study. Lancet Microbe.

[54] Basu A, Samhita L (2024). Context-dependent fitness benefits of antibiotic resistance mutations. Proc Biol Sci.

[55] Hubbard ATM, Jafari NV, Feasey N, Rohn JL, Roberts AP (2019). Effect of environment on the evolutionary trajectories and growth characteristics of antibiotic-resistant *Escherichia coli* mutants. Front Microbiol.

[56] Abdulqadir HN (2026). The plasmid-host fitness landscape: a new paradigm for predicting the fate of mobile resistance. Appl Environ Microbiol.

[57] Hubbard ATM, Mason J, Roberts P, Parry CM, Corless C (2020). Piperacillin/tazobactam resistance in a clinical isolate of *Escherichia coli* due to IS26-mediated amplification of bla_TEM-1B_. Nat Commun.

[58] Johnsen PJ, Townsend JP, Bøhn T, Simonsen GS, Sundsfjord A (2011). Retrospective evidence for a biological cost of vancomycin resistance determinants in the absence of glycopeptide selective pressures. J Antimicrob Chemother.

[59] Gottesman BS, Carmeli Y, Shitrit P, Chowers M (2009). Impact of quinolone restriction on resistance patterns of *Escherichia coli* isolated from urine by culture in a community setting. Clin Infect Dis.

[60] Sundqvist M, Geli P, Andersson DI, Sjölund-Karlsson M, Runehagen A (2010). Little evidence for reversibility of trimethoprim resistance after a drastic reduction in trimethoprim use. J Antimicrob Chemother.

[61] Graf FE, Goodman RN, Gallichan S, Forrest S, Picton-Barlow E (2024). Molecular mechanisms of re-emerging chloramphenicol susceptibility in extended-spectrum beta-lactamase-producing *Enterobacterales*. Nat Commun.

[62] Musicha P, Cornick JE, Bar-Zeev N, French N, Masesa C (2017). Trends in antimicrobial resistance in bloodstream infection isolates at a large urban hospital in Malawi (1998-2016): a surveillance study. Lancet Infect Dis.

[63] Schulz zur Wiesch P, Engelstädter J, Bonhoeffer S (2010). Compensation of fitness costs and reversibility of antibiotic resistance mutations. Antimicrob Agents Chemother.

[64] Imamovic L, Sommer MOA (2013). Use of collateral sensitivity networks to design drug cycling protocols that avoid resistance development. Sci Transl Med.

[65] Aulin LBS, Liakopoulos A, van der Graaf PH, Rozen DE, van Hasselt JGC (2021). Design principles of collateral sensitivity-based dosing strategies. Nat Commun.

[66] Podnecky NL, Fredheim EGA, Kloos J, Sørum V, Primicerio R (2018). Conserved collateral antibiotic susceptibility networks in diverse clinical strains of *Escherichia coli*. Nat Commun.

[67] Imamovic L, Ellabaan MMH, Dantas Machado AM, Citterio L, Wulff T (2018). Drug-driven phenotypic convergence supports rational treatment strategies of chronic infections. Cell.

[68] Sakenova N, Cacace E, Orakov A, Huber F, Varik V (2025). Systematic mapping of antibiotic cross-resistance and collateral sensitivity with chemical genetics. Nat Microbiol.

[69] Barbosa C, Trebosc V, Kemmer C, Rosenstiel P, Beardmore R (2017). Alternative evolutionary paths to bacterial antibiotic resistance cause distinct collateral effects. Mol Biol Evol.

[70] Kim S, Lieberman TD, Kishony R (2014). Alternating antibiotic treatments constrain evolutionary paths to multidrug resistance. Proc Natl Acad Sci USA.

[71] Li X, Liu Y, Du L, Kang Y (2020). The effect of antibiotic‐cycling strategy on antibiotic‐resistant bacterial infections or colonization in intensive care units: a systematic review and meta‐analysis. Worldviews Ev Based Nurs.

[72] Siedentop B (2024). The effect of combining antibiotics on resistance: a systematic review and meta-analysis. eLife.

[73] Lai C, Ma Z, Zhang J, Wang J, Wang J (2024). Efficiency of combination therapy versus monotherapy for the treatment of infections due to carbapenem-resistant Gram-negative bacteria: a systematic review and meta-analysis. Syst Rev.

[74] Domalaon R, Idowu T, Zhanel GG, Schweizer F (2018). Antibiotic hybrids: the next generation of agents and adjuvants against gram-negative pathogens?. Clin Microbiol Rev.

[75] Parkes AL, Bardell-Cox OA, Cain RM (2025). The state of the art in dual-acting hybrid antibiotics to combat bacterial resistance. Expert Opin Drug Discov.

[76] Koh AJJ, Thombare V, Hussein M, Rao GG, Li J (2023). Bifunctional antibiotic hybrids: a review of clinical candidates. Front Pharmacol.

[77] Webber MA, Ricci V, Whitehead R, Patel M, Fookes M (2013). Clinically relevant mutant DNA gyrase alters supercoiling, changes the transcriptome, and confers multidrug resistance. mBio.

[78] Drlica K (2003). The mutant selection window and antimicrobial resistance. J Antimicrob Chemother.

[79] Andrews JM (2001). Determination of minimum inhibitory concentrations. J Antimicrob Chemother.

[80] Gonzalez N, Sevillano D, Alou L, Cafini F, Gimenez M-J (2013). Influence of the MBC/MIC ratio on the antibacterial activity of vancomycin versus linezolid against methicillin-resistant *Staphylococcus aureus* isolates in a pharmacodynamic model simulating serum and soft tissue interstitial fluid concentrations reported in diabetic patients. J Antimicrob Chemother.

[81] Sader HS, Jones RN, Rossi KL, Rybak MJ (2009). Occurrence of vancomycin-tolerant and heterogeneous vancomycin-intermediate strains (hVISA) among Staphylococcus aureus causing bloodstream infections in nine USA hospitals. J Antimicrob Chemother.

[82] Ishak A, Mazonakis N, Spernovasilis N, Akinosoglou K, Tsioutis C (2025). Bactericidal versus bacteriostatic antibacterials: clinical significance, differences and synergistic potential in clinical practice. J Antimicrob Chemother.

[83] Pereira C, Warsi OM, Andersson DI (2023). Pervasive selection for clinically relevant resistance and media adaptive mutations at very low antibiotic concentrations. Mol Biol Evol.

[84] Bottery MJ, Wood AJ, Brockhurst MA (2016). Selective conditions for a multidrug resistance plasmid depend on the sociality of antibiotic resistance. Antimicrob Agents Chemother.

[85] Sanz-García F, Hernando-Amado S, Martínez JL (2022). Evolution under low antibiotic concentrations: a risk for the selection of *Pseudomonas aeruginosa* multidrug-resistant mutants in nature. Environ Microbiol.

[86] Firsov AA, Vostrov SN, Lubenko IY, Drlica K, Portnoy YA (2003). In vitro pharmacodynamic evaluation of the mutant selection window hypothesis using four fluoroquinolones against *Staphylococcus aureus*. Antimicrob Agents Chemother.

[87] Blondeau JM (2009). New concepts in antimicrobial susceptibility testing: the mutant prevention concentration and mutant selection window approach. Vet Dermatol.

[88] Dong Y, Zhao X, Domagala J, Drlica K (1999). Effect of fluoroquinolone concentration on selection of resistant mutants of *Mycobacterium bovis* BCG and *Staphylococcus aureus*. Antimicrob Agents Chemother.

[89] Drlica K, Zhao X (2007). Mutant selection window hypothesis updated. Clin Infect Dis.

[90] Zhang L, Wang H, Bai Y, Wang L, Bai Y (2023). Evaluation of the mutant selection window of danofloxacin against *Actinobacillus pleuropneumoniae* in an in vitro dynamic model. Front Vet Sci.

[91] Zhang N, Ye X, Wu Y, Huang Z, Gu X (2017). Determination of the mutant selection window and evaluation of the killing of *Mycoplasma gallisepticum* by danofloxacin, doxycycline, tilmicosin, tylvalosin and valnemulin. PLoS One.

[92] Feng Z-H, Fan L, Yang J, Huo X-Y, Guo Y (2019). Mutant selection window of clarithromycin for clinical isolates of *Helicobacter pylori*. BMC Microbiol.

[93] James B, Reesaul H, Kashif S, Behruznia M, Meehan CJ (2024). The effect of antibiotic selection on collateral effects and evolvability of uropathogenic *Escherichia coli*. NPJ Antimicrob Resist.

[94] Gehring R, Riviere JE (2013). Limitations of MIC as the sole criterion in antimicrobial drug dosage regimen design: the need for full characterization of antimicrobial pharmacodynamic profile especially for drug-resistant organisms. Vet J.

[95] Magréault S, Jauréguy F, Carbonnelle E, Zahar J-R (2022). When and how to use mic in clinical practice?. Antibiotics.

[96] Mouton JW, Muller AE, Canton R, Giske CG, Kahlmeter G (2018). MIC-based dose adjustment: facts and fables. J Antimicrob Chemother.

[97] Alexander HK, MacLean RC (2020). Stochastic bacterial population dynamics restrict the establishment of antibiotic resistance from single cells. Proc Natl Acad Sci USA.

[98] Goldstein I, Ehrenreich IM (2021). The complex role of genetic background in shaping the effects of spontaneous and induced mutations. Yeast.

[99] Luo N, Pereira S, Sahin O, Lin J, Huang S (2005). Enhanced in vivo fitness of fluoroquinolone-resistant *Campylobacter jejuni* in the absence of antibiotic selection pressure. Proc Natl Acad Sci U S A.

[100] Hoeksema M, Jonker MJ, Brul S, Ter Kuile BH (2019). Effects of a previously selected antibiotic resistance on mutations acquired during development of a second resistance in *Escherichia coli*. BMC Genomics.

[101] Bottery MJ, Pitchford JW, Friman V-P (2021). Ecology and evolution of antimicrobial resistance in bacterial communities. ISME J.

[102] Perlin MH, Clark DR, McKenzie C, Patel H, Jackson N (2009). Protection of *Salmonella* by ampicillin-resistant Escherichia coli in the presence of otherwise lethal drug concentrations. Proc Biol Sci.

[103] Sorg RA, Lin L, van Doorn GS, Sorg M, Olson J (2016). Collective resistance in microbial communities by intracellular antibiotic deactivation. PLoS Biol.

[104] Diaz Caballero J, Wheatley RM, Kapel N, López-Causapé C, Van der Schalk T (2023). Mixed strain pathogen populations accelerate the evolution of antibiotic resistance in patients. Nat Commun.

[105] Bustamante M, Koopman F, Martens J, Brons JK, DelaFuente J (2024). Community context influences the conjugation efficiency of *Escherichia coli*. FEMS Microbes.

[106] Fait A, Angst DC, Stylianou V, Brülisauer L, Brugger SD (2026). Inhibition of multidrug-resistant *Staphylococcus aureus* by commensal bacterial species from the human nose. bioRxiv.

[107] Lindon S, Shah S, Gifford DR, Lood C, Gomis Font MA (2024). Antibiotic resistance alters the ability of *Pseudomonas aeruginosa* to invade bacteria from the respiratory microbiome. Evol Lett.

[108] Pittaccio L, Knowles J, Wheatley RMY (2026). Microbial interactions as key players shaping the emergence and spread of de novo resistance mutations. Microbiology.

[109] Eladawy M, McCartney AL, Garner AC, Hoyles L (2025). Growth of Gram-negative uropathogens in human urine under hypoxic conditions produces clinically relevant metabolomic profiles. Microbiology.

[110] Stecher B, Maier L, Hardt W-D (2013). “Blooming” in the gut: how dysbiosis might contribute to pathogen evolution. Nat Rev Microbiol.

[111] Baomo L, Lili S, Moran RA, van Schaik W, Chao Z (2022). Temperature-regulated IncX3 plasmid characteristics and the role of plasmid-encoded H-NS in thermoregulation. Front Microbiol.

[112] Sweeney E, Sabnis A, Edwards AM, Harrison F (2020). Effect of host-mimicking medium and biofilm growth on the ability of colistin to kill *Pseudomonas aeruginosa*. Microbiology.

[113] James B, Wilde MJ, Fryer MT, Murray BO, Whiley DJ (2026). In vitro evolution of uropathogenic *Escherichia coli* to fosfomycin resistance in a 3D cultured human bladder microtissue model. bioRxiv.

[114] Clarke L, Pelin A, Phan M, Wong A (2020). The effect of environmental heterogeneity on the fitness of antibiotic resistance mutations in *Escherichia coli*. Evol Ecol.

[115] Murray BO, Flores C, Williams C, Flusberg DA, Marr EE (2021). Recurrent urinary tract infection: a mystery in search of better model systems. Front Cell Infect Microbiol.

[116] Jafari NV, Rohn JL (2023). An immunoresponsive three-dimensional urine-tolerant human urothelial model to study urinary tract infection. Front Cell Infect Microbiol.

[117] Sweeney E, Harrington NE, Harley Henriques AG, Hassan MM, Crealock-Ashurst B (2021). An ex vivo cystic fibrosis model recapitulates key clinical aspects of chronic *Staphylococcus aureus* infection. Microbiology.

[118] Turner AM, Li L, Monk IR, Lee JYH, Ingle DJ (2024). Rifaximin prophylaxis causes resistance to the last-resort antibiotic daptomycin. Nature.

[119] Chen H, Sapula SA, Turnidge J, Venter H (2025). The effect of commonly used non-antibiotic medications on antimicrobial resistance development in *Escherichia coli*. NPJ Antimicrob Resist.

